# Pharmacokinetics and Intestinal Metabolism of Compound K in Rats and Mice

**DOI:** 10.3390/pharmaceutics12020129

**Published:** 2020-02-03

**Authors:** Ji-Hyeon Jeon, Bitna Kang, Sowon Lee, Sojeong Jin, Min-Koo Choi, Im-Sook Song

**Affiliations:** 1 College of Pharmacy and Research Institute of Pharmaceutical Sciences, Kyungpook National University, Daegu 41566, Korea; kei7016@naver.com (J.-H.J.); okjin917@hanmail.net (S.L.); 2 College of Pharmacy, Dankook University, Cheon-an 31116, Korea; qlcska8520@naver.com (B.K.); astraea327@naver.com (S.J.)

**Keywords:** compound K, protopanaxadiol (PPD), pharmacokinetics, biliary excretion, intestinal metabolism

## Abstract

We aimed to investigate the plasma concentration, tissue distribution, and elimination of compound K following the intravenous administration of compound K (2 mg/kg) in rats and mice. The plasma concentrations of compound K in mice were much higher (about five-fold) than those in rats. In both rats and mice, compound K was mainly distributed in the liver and underwent biliary excretion. There was 28.4% fecal recovery of compound K in mice and 13.8% in rats, whereas its renal recovery was less than 0.1% in both rats and mice. Relative quantification of compound K and its metabolite protopanaxadiol (PPD) in rat bile and intestinal feces indicated that the metabolism from compound K into PPD occurred in the intestine but not in the plasma. Therefore, PPD detected in the plasma samples could have been absorbed from the intestine after metabolism in control rats, while PPD could not be detected in the plasma samples from bile duct cannulated rats. In conclusion, mice and rats shared common features such as exclusive liver distribution, major excretion pathway via biliary route, and intestinal metabolism to PPD. However, there were significant differences between rats and mice in the plasma concentrations of compound K and the fecal recovery of compound K and PPD.

## 1. Introduction

Compound K, which belongs to the protopanaxadiol (PPD)-type ginsenoside group, was first discovered in 1972 from a hydrolase mixture of ginsenosides (Rb1, Rb2, and Rc) and soil bacteria [1]. Since then, compound K has attracted special attention among the various ginsenosides because it is reported as a major pharmacologically active component that has hepatoprotective, chemo-preventive, anti-diabetic, anti-inflammatory, anti-arthritic, neuroprotective, and immune stimulating effects [1,2]. The therapeutic benefit of compound K has been demonstrated in both in vitro studies and in vivo disease models [1]. Paek et al. [3] investigated the dose-dependent bioavailability of compound K by comparing its oral and intravenous administration in rats. The area under plasma concentration (AUC) of compound K increased linearly following intravenous injection at a dose range of 1–10 mg/kg (dose increase 10-fold; AUC increase 11.6-fold), but the AUC of compound K following oral administration did not increase linearly at a dose range of 5–20 mg/kg (dose increase four-fold; AUC increase 75.8-fold) [3]. The AUC of compound K was significantly increased (23.5-fold) in P-glycoprotein (P-gp) knock-out mice compared to wild-type mice after a single oral dose (10 mg/kg) [4]. Therefore, P-gp-mediated efflux during intestinal absorption could be a possible explanation for non-linear oral bioavailability of compound K. 

The safety, pharmacokinetics, and preliminary efficacy of compound K in tablet form as an anti-rheumatoid arthritis drug are under clinical investigation in China (Study No. NCT03755258) [5]. In this study, Chen et al. [5] investigated the pharmacokinetics of compound K and PPD, a metabolite of compound K, following a single oral administration of a 200 mg compound K tablet. This was the first pharmacokinetic study in humans using pure compound K. The maximum plasma concentration (*C*_max_) of compound K was 796.8 ng/mL with a time to reach *C*_max_ (*T*_max_) of 3.6 h. On the other hand, the *C*_max_ of PPD was 5.7 ng/mL with a *T*_max_ of 24.5 h. The results suggested that the formation of PPD from compound K occurred slowly. The AUC ratio of PPD to compound K was calculated as 0.04 [5]. They also investigated the effect of high-fat meal on the pharmacokinetics of compound K. The high-fat meal consumption increased *C*_max_ (2.0-fold) and AUC (2.2-fold) of compound K but decreased *T*_max_ (3.6 h in fasting group vs. 2.5 h in high-fat meal group, *p* < 0.05) compared with fasting group, suggesting that the high-fat meal accelerated the absorption of compound K [5]. 

Other pharmacokinetic studies of compound K have been reported in human subjects following oral administration of ginseng product [2,6,7,8,9]. The *C*_max_ of compound K was 41.5 ng/mL in 12 Japanese subjects following a single oral administration of fermented ginseng tablet (274.4 mg total; 2.2 mg as compound K) [6]. The mean *C*_max_ (254.5 ng/mL) was substantially higher and less variable in subjects who were orally administered fermented Korean red ginseng (3 g total; 10.9 mg as compound K) than the *C*_max_ (3.2–24.8 ng/mL) in subjects who received non-fermented Korean red ginseng extract (3 g total; 0 mg as compound K) [2,7,9]. The results suggested that the consumption of ginseng product with higher compound K content resulted in higher plasma concentrations of compound K. The compound K in the plasma was absorbed after the metabolism from Rb1, Rb2, Rc, and Rd (major components of red ginseng product) to compound K following oral administration of non-fermented red ginseng product [7,10]. Therefore, the variability in the gut metabolism and intestinal absorption of compound K could be attributed to the variable plasma concentrations of compound K. However, little information is available on the distribution and elimination of compound K. Therefore, the purpose of this study was to investigate the pharmacokinetics of compound K in rats and mice with a focus on tissue distribution, elimination, and metabolism to PPD.

## 2. Materials and Methods 

### 2.1. Materials

Compound K and 20(S)-protopanaxadiol (PPD) was purchased from the Ambo Institute (Daejeon, Korea). To be used as internal standards (IS), 13C-caffeine was purchased from Sigma-Aldrich Chemical Co. (St. Louis, MO, USA). All other chemicals and solvents were of reagent or analytical grade.

### 2.2. Animals and Ethical Approval

Male Institute of Cancer Research (ICR) mice (7 or 8-weeks-old, weighing 34–37 g) and male Sprague-Dawley (SD) rats (7 or 8-weeks-old, 230–270 g) were purchased from Samtako Co. (Osan, Korea). The animals were acclimatized for 1 week at an animal facility at Kyungpook National University. Food and water were available ad libitum. All animal procedures were approved by the Animal Care and Use Committee of Kyungpook National University (Approval No. KNU 2018-192, 19 December 2018) and carried out in accordance with the National Institutes of Health guidance for the care and use of laboratory animals. An overview of the study design and methods is provided in Table 1.

### 2.3. Pharmacokinetic Study

ICR mice and SD rats received compound K intravenously at a single dose of 2 mg/kg via the tail vein and were returned to the metabolic cage with water and chow ad libitum. Before administration, the compound K was dissolved in a vehicle containing DMSO: saline (2:8, *v*/*v*) (vehicle volume, 1 mL/kg for mice and 0.4 mL/kg for rats). Blood samples (approximately 100 µL) were collected at 0, 0.17, 0.5, 1, 2, 4, 8, 24, and 48 h following intravenous injection of compound K with no sign of hemoglobinemia and hemoglobinuria. Blood sampling was performed using a sparse sampling method (time schedule is given in Table 2). The blood samples were centrifuged at 16,000× *g* for 10 min to separate the plasma. An aliquot (50 µL) of each plasma sample was stored at −80 °C until the analysis. Urine and feces samples were collected from 0–24 h and 24–48 h following the compound K administration. Aliquots (50 μL) of urine and 10% feces homogenates were stored at −80 °C until the analysis.

SD rats were randomly divided into two groups: the non-bile cannulated control group and the bile cannulated group. For the bile cannulated rats, the femoral artery, femoral vein, and bile duct were cannulated with polyethylene tubes (PE-50 and PE-10; Jungdo, Seoul, Korea) under light anesthesia with isoflurane. For the control group, the femoral artery and femoral vein were cannulated with PE-50. Compound K was injected intravenously via the femoral vein at 2 mg/kg. Blood samples (approximately 200 µL) were collected from the femoral artery at 0, 0.25, 0.5, 1, 2, 4, and 8 h after the compound K injection. After each blood sampling, normal saline was injected into the femoral vein to compensate for blood loss. Bile samples were collected every 2 h for a total of 12 h. The blood samples were centrifuged at 16,000× *g* for 10 min, and 50 μL aliquots of plasma and 50 μL aliquots of bile were stored at −80 °C until the compound K analysis. In the non-bile cannulated rats, the complete contents of the entire gastrointestinal tract were collected using a 10 mL syringe filled with 30 mL pre-warmed saline. The contents were homogenized with tissue homogenizer, and 50 μL aliquots of intestinal fecal homogenates were stored at −80 °C until the analysis.

The 50 μL samples of plasma, urine, 10% fecal homogenates, bile, and intestinal fecal homogenates were each mixed with 60 μL of IS (20 ng/mL 13C-caffeine in water) and 600 μL of methyl *tert*-butyl ether (MTBE). The mixtures were vortexed vigorously for 10 min and centrifuged at 16,000× *g* for 5 min. After centrifugation, the samples were frozen at −80 °C for 2 h. For each sample, the upper layer was transferred to a clean tube and evaporated to dryness under a nitrogen stream. The residue was reconfigured with 200 μL of 80% methanol consisting of 0.1% formic acid, and a 10 μL aliquot was injected into the liquid chromatography–tandem mass spectrometry (LC–MS/MS) system. 

### 2.4. Tissue Distribution Study

ICR mice and SD rats received compound K intravenously at a single dose of 2 mg/kg via tail vein and were returned to the metabolic cage with water and chow ad libitum. The animals were euthanized and blood samples (approximately 200 µL) were collected from the abdominal artery at 0.17, 0.5, 2, 4, 8, and 24 h after intravenous injection. The liver, kidney, brain, heart, lung, spleen, and testis were immediately excised, gently washed with ice-cold saline, and weighed. The tissue samples were homogenized with 4 volumes of saline. Aliquots (50 μL) of plasma and 20% tissue homogenates were stored at –80 °C until the analysis of compound K. 

To investigate the effect of rifampin (a representative inhibitor of the organic anion-transporting polypeptide (Oatp) transporters [11]) on the hepatic distribution of compound K, ICR mice and SD rats were randomly divided into either control or rifampin groups. The rifampin group was orally administered with rifampin solution (20 mg/kg, dissolved in DMSO: saline = 2:8, *v*/*v*) and the control group received only the vehicle via oral gavage. One hour after rifampin treatment, mice and rats received compound K intravenously at a single dose of 2 mg/kg via tail vein. The animals were euthanized and blood samples (approximately 200 µL) were collected from the abdominal artery at 0.5 and 2 h after intravenous injection. The liver tissues were immediately excised, gently washed with ice-cold saline, and homogenized with 4 volumes of saline. Aliquots (50 μL) of plasma and 20% liver homogenates were stored at −80 °C until the analysis of compound K. 

The plasma samples and 20% tissue homogenates samples were each mixed with 60 μL of IS (20 ng/mL 13C-caffeine in water) and 600 μL of MTBE. The mixtures were vortexed vigorously for 10 min and centrifuged at 16,000× *g* for 5 min. After centrifugation, the samples were frozen at −80 °C for 2 h. The upper layer was transferred to a clean tube and evaporated to dryness under a nitrogen stream. The residue was reconfigured with 200 μL of 80% methanol consisting of 0.1% formic acid, and a 10 μL aliquot was injected into the LC-MS/MS system. 

### 2.5. LC-MS/MS Analysis of Compound K

Compound K and PPD concentrations were analyzed using a modified LC-MS/MS method of Jin et al. [8] with an Agilent 6430 triple quadrupole LC-MS/MS system (Agilent, Wilmington, DE, USA). Compound K and PPD were separated using an Eclipse Plus C18 RRHD (1.8 µm particle size, 3.0 × 5.0 mm, Agilent, Wilmington, DE, USA). The mobile phase consisted of 0.1% formic acid in water (8%) and 0.1% formic acid in methanol (92%) at a flow rate of 0.15 mL/min.

Quantification of a separated analyte peak was performed at *m*/*z* 645.5 → 203.1 for compound K (*T*_R_ (retention time) 6.9 min), *m*/*z* 425.3 → 109.1 for PPD (*T*_R_ 13.9 min), and *m*/*z* 198.2 → 140.1 for 13C-caffeine (IS) (*T*_R_ 2.9 min) in the positive ionization mode with collision energy (CE) of 35, 25 and 20 eV, respectively. The analytical data were quantified using Mass Hunter (version B.06.00, Agilent, Wilmington, DE, USA).

The calibration standards and quality control (QC) samples were prepared by spiking a 5 µL aliquot of the working solution with 45 µL aliquot of blank matrix (plasma, liver, kidney, heart, lung, pancreas, brain, testis, and urine). The final concentrations of the compound K and PPD calibration standards for plasma and urine samples were 5, 10, 20, 50, 200, 500, 2000 ng/mL, and the concentrations of QC samples of compound K and PPD were 15, 100, and 1500 ng/mL. The concentrations of calibration standards and QC samples of compound K and PPD for bile and fecal homogenates were 25, 50, 100, 250, 1000, 2500, and 10,000 ng/mL and 75, 500, and 7500 ng/mL, respectively. The concentrations of calibration standards and QC samples of compound K for liver, kidney, heart, lung, pancreas, brain, and testis homogenates were 5, 10, 20, 50, 200, 500, and 2000 ng/mL and 15, 100, and 1500 ng/mL, respectively. The standard calibration curves for compound K and PPD was linear in the concentration range of 5–2000 ng/mL in the plasma, urine, and tissue homogenates samples and in the concentration range of 25–10,000 ng/mL in the bile and feces homogenates samples, respectively. The inter-day and intra-day precision and accuracy (%CV) for compound K and PPD in all biological samples was less than 15%. 

### 2.6. Data Analysis

Pharmacokinetic parameters were estimated using non-compartmental methods (WinNonlin version 2.0, Pharsight Co., Certara, NJ, USA). 

All pharmacokinetic parameters are given as the mean ± standard deviation. All statistical analyses were performed using SAS (ver. 9.4; SAS Institute Inc., Cary, NC, USA). A *p*-value < 0.05 was considered statistically significant.

## 3. Results

### 3.1. Comparative Pharmacokinetics of Compound K in Rats and Mice

**Figure 1:**
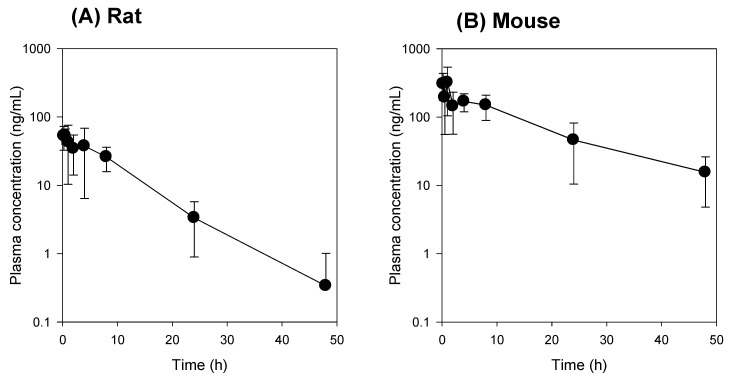
(**A**) Plasma concentration vs. time profile of compound K following intravenous injection of compound K at a single dose of 2 mg/kg in rats (**A**) and mice (**B**). Plasma concentration of compound K (*Y*-axis) was represented using a logarithmic scale. Data expressed as mean± standard deviation from four rats or four mice at different time points.

The plasma concentration-time profile of compound K was compared between the mice and the rats. Since the intestinal absorption of compound K is low and variable [3,4], intravenous injection of compound K was used in this study intead of oral administration. The plasma concentrations of compound K in mice were greater than those in rats (Figure 1). The pharmacokinetic parameters of compound K such as AUC and plasma concentration were about 5–6-fold greater in mice than in rats, while there was no significant difference in the half-life (*T*_1/2_) and mean residence time (MRT) between rats and mice (Table 3). However, the clearance (CL) and volume of distribution (Vd) values were about five-fold larger in rats than in mice (Table 3). Taken together, the results suggest that the distribution and elimination of compound K differ between rats and mice. 

To compare the elimination pathway of compound K, we measured the renal and fecal recovery of compound K following intravenous administration of compound K in both rats and mice. The recovery of compound K from the urine was about 0.02% of the intravenous dose in both mice and rats. In contrast, the fecal recovery of compound K was about 28.4 ± 5.9% in mice and 13.8 ± 7.1% in rats (Table 1). The results suggest that fecal excretion is a major excretion route for compound K in both rats and mice but also that compound K may undergo in vivo metabolism in both rats and mice.

### 3.2. Tissue Distribution of Compound K

**Figure 2:**
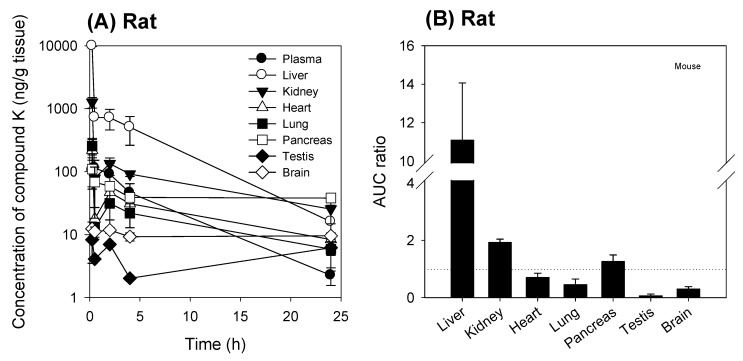
(**A**) Tissue concentration vs. time profile of compound K following intravenous injection of compound K at a single dose of 2 mg/kg in rats. (**B**) AUC ratios (AUC_tissue_/AUC_plasma_) of compound K in the liver, kidney, heart, lung, pancreas, testis, and brain following intravenous injection (2 mg/kg) in rats. AUC was calculated from the data shown in (**A**) and the dotted line in (**B**) represents unity. Data are expressed as mean± standard deviation from four rats at different time points.

**Figure 3:**
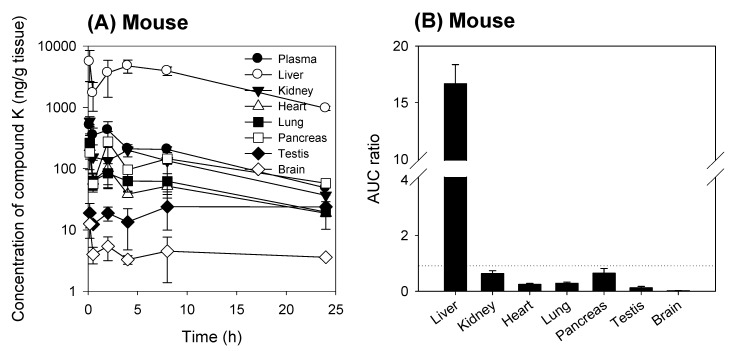
(**A**) Tissue concentration vs. time profile of compound K following intravenous injection of compound K at a single dose of 2 mg/kg in mice. (**B**) AUC ratios of compound K in the liver, kidney, heart, lung, pancreas, testis, and brain to plasma following intravenous injection (2 mg/kg) in mice. AUC was calculated from the data shown in (**A**) and the dotted line in (**B**) represents unity. Data are expressed as mean ± standard deviation from four rats at different time points.

To analyze the tissue distribution of compound K, we measured the temporal profile of compound K in various tissues in rats and mice following intravenous administration of compound K. As shown in Figure 2 and Figure 3, the tissue distribution pattern of compound K was comparable in both mice and rats. Compound K was predominantly distributed to the liver in both rats and mice, and the liver to plasma AUC ratio was 11.1 in rats and 16.7 in mice. The AUC ratios of other tissues (i.e., kidney, heart, lung, pancreas, testis, and brain) to plasma, however, were much lower than the liver/plasma AUC ratio for both species (Figure 2B and Figure 3B). These results suggest the involvement of the uptake transport system, which is dominantly expressed in the liver. Previously, Jiang et al. [12] reported the involvement of the Oatp transporters (OATP1B3 in humans and Oatp1b2 in rats) in the hepatic uptake of ginsenosides Rg1, Re, and R1. Therefore, we investigated the effect of rifampin, a representative inhibitor of the Oatp transporter [11], on the liver distribution of compound K to further assess the involvement of the Oatp uptake transporter.

**Figure 4:**
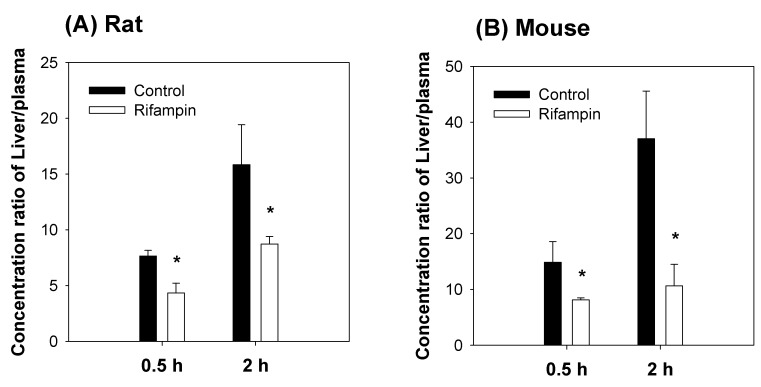
Effect of rifampin pre-treatment on the liver to plasma concentration ratios of compound K in (**A**) rats and (**B**) mice. Liver and plasma compound K concentration were measured following intravenous injection of compound K at a single dose of 2 mg/kg in the presence or absence of rifampin pre-treatment (20 mg/kg, per oral). Data expressed as mean ± standard deviation from four rats or four mice at different time points. *: *p* < 0.05 compared with control group.

When rifampin was given orally at 1 h prior to compound K injection, the plasma concentration of compound K increased and the liver to plasma concentration ratios decreased when compared to the control group (no rifampin pre-treatment) in both mice and rats (Figure 4). This can be explained by the blocking of Oatp-mediated hepatic uptake of compound K by the rifampin pre-treatment. 

### 3.3. Intestinal Metabolism of Compound K

**Figure 5:**
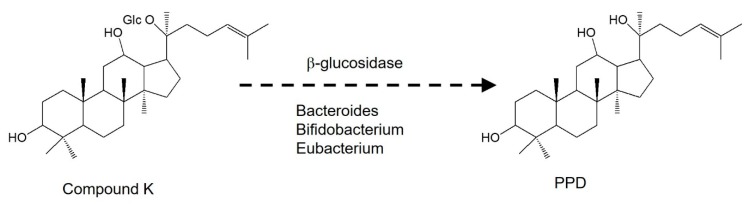
Structure and reported metabolic pathway of compound K to PPD. Glc: glucose; PPD: 20(S)-protopanaxadiol.

**Figure 6:**
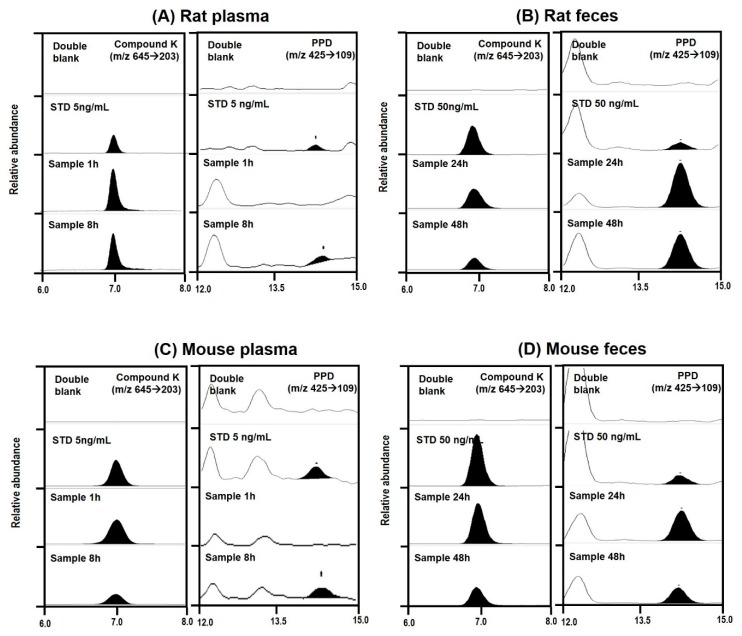
Representative multiple reaction monitoring chromatogram of compound K and PPD in plasma and feces samples collected from rats (**A**,**B**) and mice (**C**,**D**) following intravenous injection of compound K at a dose of 2 mg/kg.

To understand the metabolism of compound K, we measured PPD levels in the plasma, urine, and feces samples from rats and mice. Previous studies have demonstrated that compound K is metabolized to PPD via β-glucosidase in intestinal microbacterium (Figure 5) [1,13]. As shown in Figure 6, PPD was detected in the plasma and fecal samples from both mice and rats following intravenous injection of compound K (2 mg/kg). However, PPD was not detected in the rat and mouse urine samples. These results suggest that compound K was metabolized to PPD in both rats and mice. 

**Figure 7:**
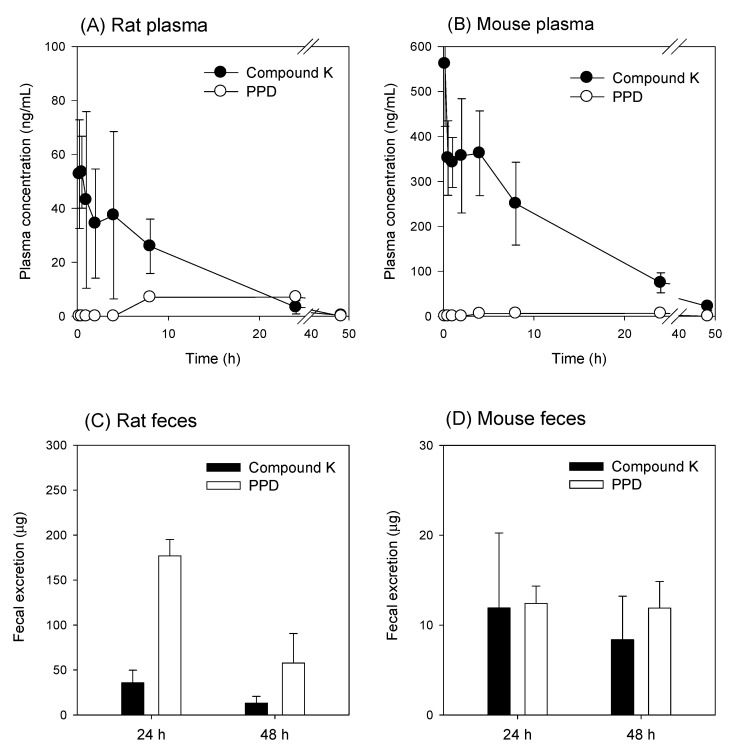
Plasma concentration vs. time profile of compound K and PPD (a metabolite of compound K) following intravenous injection of compound K at a single dose of 2 mg/kg in (**A**) rats and (**B**) mice. Fecal excretion of compound K and PPD following intravenous injection of compound K at a single dose of 2 mg/kg in (**C**) rats and (**D**) mice. Data expressed as mean± standard deviation of four samples for each time point.

The concentrations of compound K and PPD in plasma and feces samples are shown in Figure 7. Following intravenous injection of compound K, the PPD peak was detected in the mouse plasma samples between 4 and 24 h and in the rat plasma samples between 8 and 24 h (Figure 7A,B), suggesting that the metabolism of compound K to PPD might be a slow process. The PPD concentration in the plasma samples at 48 h was below the lower limit of quantification (LLOQ; 5 ng/mL). The metabolic ratio calculated from the PPD to compound K ratio was much greater in rat plasma than in mouse plasma (0.02–0.08 in mouse vs. 0.2–1.6 in rat; Figure 7A,B). Similarly, the ratio of PPD to compound K in the feces samples was also significantly greater in rats than in mice (Figure 7C,D). The recovery of PPD was 3.4-fold higher than that of compound K in rat feces, whereas the recovery of PPD was 1.2-fold higher than that of compound K in mouse feces. As shown in Table 4, compound K and its metabolite PPD were exclusively recovered from feces but not from urine. The higher percentage of PPD recovery in rats compared to mice suggest the higher metabolism of compound K to PPD in rats compared to mice (Figure 1 and Table 3).

**Figure 8:**
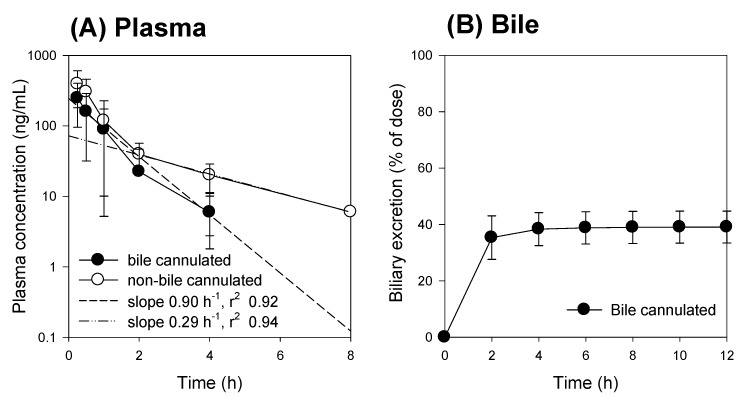
(**A**) Plasma concentration and (**B**) biliary excretion of compound K following intravenous injection at a single dose of 2 mg/kg in the bile-cannulated rats (●) and in the non-bile-cannulated rats (○). Dotted lines represent the regression line of the elimination constant from the plasma concentrations of compound K. Data expressed as mean ± standard deviation from four rats per group.

**Figure 9:**
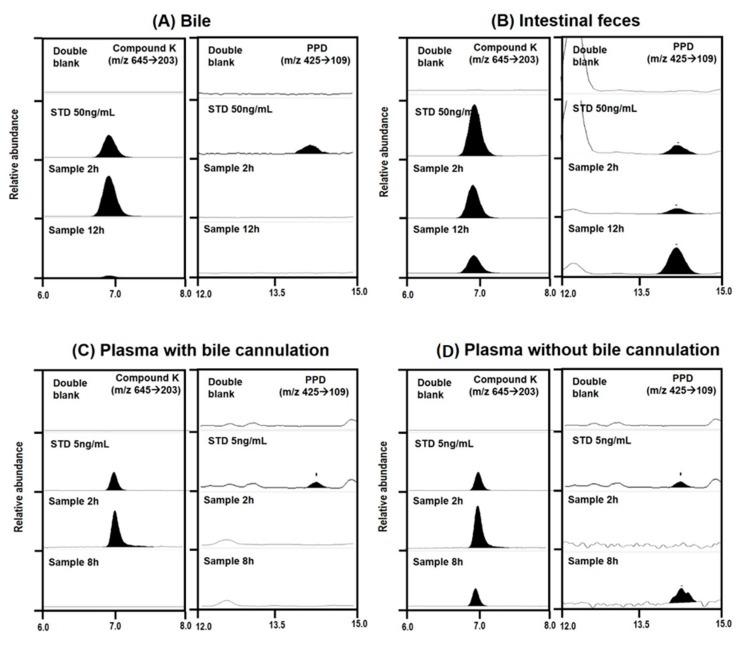
Representative multiple reaction monitoring chromatogram of compound K and PPD in (**A**) bile collected for 0–2 h and 2–12 h from bile cannulated rats, (**B**) intestinal feces samples collected from rats of non-bile cannulation at 2 or 12 h, (**C**) plasma samples taken at 2 and 8 h from bile cannulated rats, and (**D**) plasma samples taken at 2 and 8 h from rats of non-bile cannulation following intravenous injection of compound K (2 mg/kg).

Next, we compared the pharmacokinetic features of compound K between the bile-cannulated rats and the non-bile-cannulated rats. In the bile-cannulated rats, biliary excretion of compound K was very fast and most of the excreted compound K was collected during the 0–2 h period (Figure 8B), which could be due to high and fast distribution of compound K to the liver (Figure 2). Because of the fast distribution to the liver and biliary excretion, the plasma concentration of compound K disappeared rapidly and was best fitted to the one-compartment model. The elimination constant was estimated using 5 points and yielded 0.90 h^−1^ with an *r*^2^ value of 0.92 (Figure 8A). The T_1/2_ was calculated as 0.78 ± 0.11 h in the bile-cannulated rats. In contrast, the AUC value for compound K in the non-bile-cannulated rats was significantly greater than that of the non-bile cannulated control rats (Table 5). The plasma concentration profile of compound K in the control group showed 2-exponential decay. The elimination constant was estimated using 3 points and yielded 0.29 h^−1^ with *r*^2^ value of 0.94 (Figure 9A). As results, *T*_1/2_ was calculated as 2.40 ± 0.61 h in the non-bile cannulated rats, which is significantly greater than that in bile cannulated rats. The results suggest that the compound K enters into the systemic circulation from other compartments.

We compared the compound K and PPD levels in the plasma and bile or intestinal feces samples in the bile duct cannulated rats and the control rats (without bile duct cannulation) to determine whether biotransformation from compound K to PPD occurred in the intestine or the plasma. For this, bile samples were collected for 0–2 h and 2–12 h period after administration of compound K from the rats with bile duct cannulation. Since these bile samples were collected directly from the bile duct, the compound K in the sample was distributed to the liver and excreted through the biliary route and, therefore, did not reach the intestinal microbiota. In control group, however, the compound K in the bile sample was excreted via the bile duct, so it reached the intestine and was subjected to further metabolism to PPD. As shown in Figure 8, all of the bile samples collected over a 12 h period from the bile-cannulated rats contained only compound K without PPD (Figure 9A), suggesting no further metabolism of compound K to PPD occurred in the rat plasma, liver, and bile. However, the intestinal fecal samples collected from rats without bile cannulation at 2 and 12 h following intravenous injection of compound K showed peaks for both compound K and PPD, and the amount of PPD was greater in the 12 h samples compared to the 2 h samples (Figure 9B). In addition, PPD was detected in the plasma samples at 8 h in the control rats (Figure 9D), suggesting that the PPD detected in the 8 h sample (which was not present in the 2 h plasma sample) was absorbed from the intestine after metabolism of compound K occurred. These results were consistent with the results from Figure 7A. Contrary to the control group, PPD was not detected in any of the plasma samples collected from bile-cannulated rats (Figure 9C). Taken together, the results suggest slow biotransformation of compound K to PPD in the rat intestine followed by the reabsorption of PPD into the systemic circulation.

## 4. Discussion

Although much is known about the pharmacological effects of compound K from in vitro studies and in vivo disease models, research on the pharmacokinetics as well as the relationship between the pharmacokinetics and drug response of compound K has been limited. This study aimed to understand the pharmacokinetic features of compound K and to compare its pharmacokinetic behavior in rats and mice, which are often used for disease models. Our study found that biliary excretion of compound K is a major elimination pathway, and fast and extensive liver distribution of compound K was demonstrated in both rats and mice. Oatp transporter-mediated hepatic uptake could be a possible mechanism for the dominant liver distribution compared to other tissues such as kidney, brain, spleen, and testis in both species (Figure 2 and Figure 3), as the hepatic uptake of compound K was significantly inhibited by the pretreatment of rifampin, an Oatp inhibitor (Figure 4). Our results indicate that biotransformation of compound K to PPD occurs in the intestine rather than in the plasma or the liver, based on the comparison between the non-bile-cannulated rats and the bile-cannulated rats (Figure 9). The plasma PPD in rats and mice was then be reabsorbed from the intestine after the metabolism of the excreted compound K (Figure 1 and Figure 9). In addition, the higher plasma AUC and *T*_1/2_ of compound K in the non-bile cannulated rats compared with those in bile cannulated rats (Table 5) suggests that the excreted compound K was reabsorbed from the intestinal lumen. The *T*_1/2_ of compound K in non-bile cannulated rats was significantly greater (2.40 ± 0.61 h) than in the bile-cannulated rats (0.78 ± 0.11 h). In addition, the *T*_1/2_ of compound K in the control rats calculated from the plasma concentration profiles for 48 h was 7.3 ± 0.4 h (Table 3), which is much longer than the T_1/2_ calculated from the plasma concentration profiles for 12 h. Also, the compound K plasma concentrations in the bile-cannulated rats could be fitted to a 1-compartment model while the plasma concentrations in the non-bile cannulated control rats showed 2-exponential decay (Figure 8A). Moreover, the compound K plasma concentrations increased or maintained at 2–4 h after sharply decreasing in the 0–2 h period, and then showed a slow decrease over the 4–48 h time period in both rats and mice (Figure 7A,B). This pattern could be attributable to the continuous reabsorption of compound K. The lipophilicity (LogP value 3.85 for compound K; 5.53 for PPD) and moderate permeability (0.5–2 × 10^−6^ cm/s for compound K; 1.15 × 10^−6^ cm/s for PPD) of compound K and PPD in Caco-2 cells also support the possibility of compound K and PPD reabsorption [3,7,14,15]. 

The features that differed most significantly between the rats and the mice were the higher plasma concentration (C_0_ and AUC) of compound K in mice and the greater fecal recovery of PPD in rats. The percent recovery of the parent form (compound K) in mouse feces was much higher than in rat feces (13.8% in rats vs. 28.4% in mice), while the total fecal recovery (sum of compound K and PPD) was similar for both mice and rats (60.4% in rats vs. 62.8% in mice), suggesting that the elimination process of compound K could differ between rats and mice in addition to the difference in Vd between rats and mice. Multiple previous studies have shown that the tri- or four-glycosylated PPD-type ginsenosides (major components in red ginseng; Rb1, Rb2, Rc, and Rd) have been metabolized to compound K (monoglycosylated PPD-type ginsenoside) and further hydrolyzed to PPD, the final metabolite of the PPD-type ginsenosides, in the presence of lactic acid bacteria and gut microbiota [13,16]. Previous studies have reported that Bacteroides sp., Eubacterium sp., and Bifidobacterium sp. could potentially be involved in compound K metabolism and that subjects who have a higher composition of Bacteroides sp., Eubacterium sp., and Bifidobacterium sp. strains showed higher metabolism of Rb1 to compound K [1,10]. Similarly, differences in the composition of the intestinal microbiota in rats and mice could lead to the different rates of metabolism of compound K to PPD. Wang et al. identified the predominant bacterium in human and animal fecal samples [17]. In human fecal samples, 55% of colonies were identified as Bacteroides sp. but Eubacterium sp. and Bifidobacterium sp. were also present in smaller proportions. In mice and rat fecal samples, Bacteroides sp. showed relatively low expression compared to the human samples. Eubacterium sp. and Bifidobacterium sp. also showed lower expression than other species (Clostridium, Fusobacterium, and Peptosreptococcus sp.). The data suggests that there are species-dependent factors that play a role in the gut metabolism of ginsenosides in mice, rats, and humans. Kim et al. [10] reported that human subjects who have a higher proportion Bacteroides sp. in their fecal microbiota showed 6-fold higher metabolic activity of compound K than the subject group that had a smaller proportion of Bacteroides sp. Choi et al. [7] reported that inter-subject variability in gut metabolism of compound K rather than the intestinal absorption of compound K may contribute to the large inter-individual variations in plasma compound K concentrations. Therefore, differences in the gut metabolism of compound K could also explain the variability of the compound K pharmacokinetics between species. In addition, cytochrome P450 3A-mediated metabolism of PPD has been reported in human plasma and urine samples. Multiple oxidized PPD metabolites were identified from human plasma and urine samples, and cytochrome P450 3A is thought to be involved in this process [18,19]. This metabolism of PPD in the liver microsomes could explain the unrecovered portion of compound K and PPD at 48 h following intravenous administration of compound K in this study.

**Figure 10:**
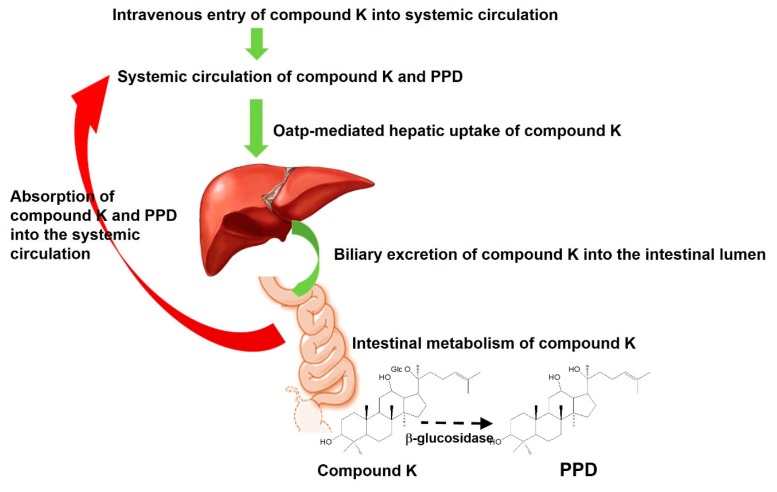
Proposed pharmacokinetic pathway of compound K following intravenous injection. Compound K underwent several steps: (i) intravenous entry of compound K into systemic circulation, (ii) Oatp-mediated hepatic uptake of compound K, (iii) biliary excretion of compound K into the intestinal lumen, (iv) metabolism of compound K into PPD in intestine, and (v) the absorption of compound K and PPD from intestine in blood.

Collectively, the proposed enterohepatic circulation of compound K and PPD (Figure 10) could explain how compound K shows efficacy in vivo despite its fast and exclusive biliary excretion. The distribution of compound K into the liver could be a possible link to the hepatoprotective effect of compound K. However, the therapeutic use of compound K in other tissues and the oral administration of compound K and PPD may be limited because of poor aqueous solubility (33 μg/mL for compound K; <50 ng/mL for PPD) and P-gp-mediated efflux [4,14,15]. The use of nanocrystals for PPD formulation improved oral bioavailability and brain delivery [15]. The use of the metabolism inhibitor piperine [20] in the formulation of PPD and the use of P-gp inhibitor, α-Tocopheryl polyethylene glycol 1000 succinate (TPGS) [21], in the formulation of PPD and compound K enhanced oral absorption and anticancer efficacy of these PPD and compound K [22,23]. These approaches may provide a strategy for developing formulations for compound K and PPD by modulating their pharmacokinetic features.

## 5. Conclusions

This comparative pharmacokinetic and tissue distribution study of compound K in rats and mice demonstrated the following processes (Figure 10). First, the plasma concentration of compound K in mice was significantly greater than that in rats and the fecal recovery of PPD over 48 h in rats was greater than that in mice although the differences in elimination and metabolism between the two species need further investigation. Second, the plasma compound K was quickly distributed into the liver and underwent biliary excretion rather than renal elimination. The distribution of compound K into other major organs (kidney, heart, lung, pancreas, and testis) was much lower than in the liver for both rats and mice. Third, compound K was metabolized into PPD by the intestinal microbiota and the intestinal PPD metabolite was reabsorbed in the systemic circulation of both rats and mice.

## Figures and Tables

**Table 1:** Overview of the study design and methods.

Study Name	SD Rats	ICR Mice	Remarks
Pharmacokinetics of compound K (*n* = 4)	Compound K IV 2 mg/kg Blood sampling: 0.17–48 h Feces collection for 48 h Urine collection for 48 h	Compound K IV 2 mg/kg Blood sampling: 0.17–48 h Feces collection for 48 h Urine collection for 48 h	Comparison between rats and mice PK parameters of compound K Fecal and urine recovery of compound K and PPD
Tissue distribution of compound K (*n* = 4)	Compound K IV 2 mg/kg Blood and tissue collection: 0.17–24 h Liver, kidney, brain, heart, lung, spleen, testis	Compound K IV 2 mg/kg Blood and tissue collection: 0.17–24 h Liver, kidney, brain, heart, lung, spleen, testis	Comparison between rats and mice Tissue to plasma AUC ratios of compound K
Inhibition of hepatic uptake of compound K (*n* = 3)	Compound K IV 2 mg/kg Rifampin pretreatment PO 20 mg/kg Blood and liver collection at 0.5 and 2 h	Compound K IV 2 mg/kg Rifampin pretreatment PO 20 mg/kg Blood and liver collection at 0.5 and 2 h	Comparison between groups with/without rifampin pretreatment Tissue to plasma concentration ratios of compound K
Biliary excretion of compound K (*n* = 4)	Compound K IV 2 mg/kg Blood sampling: 0.25–8 h Bile collection: for 12 h		PK parameters of compound K Biliary excretion of compound K
Metabolism of compound K (*n* = 4)	Compound K IV 2 mg/kg Bile collection for 0-2 h and 2–12 h Intestinal feces collection at 2 and 12 h		PPD quantification in plasma, bile, and intestinal feces samples

IV, intravenous injection; PO, per oral administration, PPD, 20(S)-protopanaxadiol; PK parameters, pharmacokinetic parameters; AUC, area under plasma concentration.

**Table 2:** Blood sampling schedule for the pharmacokinetic study of compound K in mice and rats following intravenous injection of compound K.

Sampling Time (h)	Group 1 (*n* = 4)	Group 2 (*n* = 4)	Group 3 (*n* = 4)	Sampling Method	Anesthesia
0	O			RO-right ^1^	O
0.17		O		RO-right	O
0.5			O	RO-right	O
1	O			RO-left ^2^	O
2		O		RO-left	O
4			O	RO-left	O
8	O			AA ^3^	O
24		O		AA	O
48			O	AA	O

^1^ RO-right: retro-orbital blood sampling—right eye under anesthesia with isoflurane; ^2^ RO-left: retro-orbital blood sampling—left eye under anesthesia with isoflurane; ^3^ AA: abdominal artery blood sampling.

**Table 3:** Pharmacokinetic parameters of compound K in rat and mouse.

Characteristics	Parameters	Rat	Mouse
Plasma	*C*_0_ (ng/mL)	75.3 ± 23.3	400 ± 137 *
	AUC_48 h_ (ng∙h/mL)	638.8 ± 298.6	3756.3 ± 1636 *
	AUC_∞_ (ng∙h/mL)	670.1 ± 284.1	4025.1 ± 1836 *
	*T*_1/2_ (h)	7.3 ± 0.4	11.4 ± 1.5
	MRT (h)	8.9 ± 0.4	11.8 ± 2.0
	CL (mL/min/kg)	59.0 ± 21.8	10.4 ± 4.9 *
	Vd (L/kg)	31.8 ± 3.1	7.08 ± 2.6 *
Excretion	Feces_48 h_ (% of dose)	13.8 ± 7.1	28.4 ± 5.9 *
	Urine_48 h_ (% of dose)	0.01 ± 0.01	0.02 ± 0.01

AUC_48 h_ or AUC_∞_: area under the plasma concentration-time curve from 0 to 48 h or to infinity; *C*_0_: initial plasma concentration; *T*_1/2_: half-life; MRT: mean residence time; CL: clearance; Vd: Volume of distribution. Data expressed as mean ± standard deviation (*n* = 4). *: *p* < 0.05 compared with the rat group.

**Table 4:** Recovery of compound K and PPD from urine and feces over the 48 h period following intravenous injection of compound K at a single dose of 2 mg/kg.

Species	Compounds	Recovery_48 h_ (% of Dose)	
		Feces	Urine
Rat	Compound K	13.8 ± 7.1	0.05 ± 0.03
	PPD	46.6 ± 4.9	ND
Mouse	Compound K	28.4 ± 5.9	0.02 ± 0.01
	PPD	34.4 ± 5.8	ND

ND: Not detected. Data expressed as mean ± standard deviation from four rats and mice per group.

**Table 5:** Pharmacokinetic parameters of compound K in rats of bile cannulation and non-bile cannulation.

Characteristics	Parameters	Non-Bile Cannulated Rat	Bile Cannulated Rat
Plasma	*C*_0_ (ng/mL)	451.13 ± 192.5	436.25 ± 325.8
	AUC_12 h_ (ng∙h/mL)	496.38 ± 253.1	211.09 ± 87.32 *
	AUC_∞_ (ng∙h/mL)	517.19 ± 248.6	234.78 ± 125.6 *
	*T*_1/2_ (h)	2.40 ± 0.61	0.78 ± 0.11 *
Excretion	Bile_12 h_ (% of dose)	-	39.1 ± 5.7

*C*_0_: initial plasma concentration; AUC_12 h_ or AUC_∞_: area under the plasma concentration-time curve from 0 to last sampling time or infinity; *T*_1/2_: half-life. Data expressed as mean ± standard deviation from four rats. *: *p* < 0.05 compared with non-bile cannulated rat group.
